# Real-time Technical Support Using a Remote Technology During Cardiac Implantable Electronic Device Follow-up: A Preliminary Multicenter Experience in Clinical Practice

**DOI:** 10.19102/icrm.2024.15114

**Published:** 2024-11-15

**Authors:** Valter Bianchi, Maria Silvia Negroni, Domenico Pecora, Giovanni Bisignani, Giuseppe Damiano Sanna, Stefano Nardi, Manuela Azzara, Carmelo La Greca, Concetta Torchia, Gavino Casu, Luigi Argenziano, Monica Campari, Sergio Valsecchi, Antonio D’Onofrio

**Affiliations:** 1 “Unità Operativa di Elettrofisiologia, Studio e Terapia delle Aritmie,” Monaldi Hospital, Naples, Italy; 2 Ospedale Civile, Vigevano, Italy; 3 Fondazione Poliambulanza, Brescia, Italy; 4 Castrovillari Hospital, Cosenza, Italy; 5 Clinical and Interventional Cardiology, Sassari University Hospital, Sassari, Italy; 6 Ospedale Evangelico “Villa Betania,” Naples, Italy; 7 Pineta Grande Hospital, Castel Volturno, Italy; 8 Boston Scientific Italia, Milan, Italy

**Keywords:** Cardiac implantable electronic devices, implantable cardioverter-defibrillator, remote support, subcutaneous implantable cardioverter-defibrillator screening, telemedicine

## Abstract

Industry-employed allied professionals (IEAPs) provide technical assistance to physicians during cardiac implantable electronic device (CIED) implantation, programming, troubleshooting, and follow-up. The Heart Connect™ application (Boston Scientific Inc., Marlborough, MA, USA) is a data-sharing system that enables remote access and display sharing of the CIED Programmer. This report aims to describe the preliminary experience of remote IEAP support through the application during CIED follow-up in clinical practice. The application was downloaded on the programmer, and network connections were established and tested at six Italian centers. Staff members were trained and online meetings were scheduled with IEAPs during consecutive CIED follow-up visits. Data and user feedback were collected. A total of 20 operators received training, and online meetings were conducted during 208 patient visits. Of these, 202 (97%) visits were successfully completed with remote support, without the need for additional medical or technical assistance. The connection quality, audio, and video were rated as good or excellent in ≥95% of sessions. The average duration of online meetings ranged from 6–16 min, depending on the supported session type. Comprehensive CIED checks and tests were performed during the visits, leading to the identification of relevant conditions or programming changes in 29% of visits. All operators found the application to be user-friendly and effective. Overall, satisfaction with the remote support service was rated high in 80% of responses, particularly for managing unscheduled CIED follow-up visits. In conclusion, remote support during CIED follow-up appears to be feasible, effective, and well accepted. It offers a viable alternative to traditional on-site IEAP support for both scheduled and unscheduled follow-up visits.

## Introduction

Cardiac implantable electronic devices (CIEDs) are established treatments for a variety of cardiac arrhythmias. Implantable cardioverter-defibrillators (ICDs) have long been recommended in clinical guidelines for treating ventricular arrhythmias^1^ and managing chronic heart failure frequently in association with cardiac resynchronization therapy (CRT-D).^2^ Traditionally, industry-employed allied professionals (IEAPs) have played a crucial role in providing technical assistance to physicians during pacemaker/ICD implantation, programming, troubleshooting, and follow-up.^3^ The presence of device-specific algorithms, unique to each CIED manufacturer, can pose challenges for implanters and clinic staff, requiring guidance on optimal programming to enhance device efficacy and safety and minimize the need for future reprogramming. As the prevalence of cardiovascular disease increases, there is a growing demand for technical assistance. Virtual care for cardiology services offers a more convenient and cost-effective approach to managing patients with cardiovascular disease. The Heart Connect™ application (Boston Scientific Inc., Marlborough, MA, USA) is a data-sharing system designed to facilitate online meetings between physicians and IEAPs, allowing them to remotely access and share the display of the CIED Programmer. This system serves as a remote access solution to provide prompt support to CIED centers, offering quick feedback on pre- and post-procedural device programming, patient follow-up, and faster troubleshooting. The adoption of this solution is expected to enhance hospital productivity, reduce service costs, and minimize travel expenses.

This report aims to describe the preliminary experience of using remote IEAP support by means of the Heart Connect™ application during CIED follow-up in clinical practice.

## Methods

### Evaluation design

Starting in October 2022, the Heart Connect™ software was downloaded on the programmer, and a network connection was established and tested using both Wi-Fi and a 4G cellular adapter at six Italian arrhythmia centers. An external headset and a webcam were connected to the programmer. After the system was set up, the staff members were briefly trained on how to use the application.

During consecutive follow-up visits, no in-person support from IEAPs was provided, but online meetings were established with IEAPs of the Remote Clinical Support team at the beginning of each CIED follow-up assessment. The visit was carried out by the center’s health care personnel (cardiologist, nurse, or internal technician) in accordance with international recommendations^4^ and under local standard-of-care conditions of use. The content of the assessment depended on clinical and technical factors and upon the type of CIED. If relevant conditions were identified or actions or programming changes were done during the visit, they were documented. The Heart Connect™ system was also used to provide remote support during sessions of subcutaneous ICD (S-ICD) eligibility verification before implantation, through the surface electrocardiogram (ECG) automated screening tool.^5^ Data and users’ feedback were collected on the quality of the remote connection and the support at each meeting. In May 2023, the operators were asked to reply to an ad hoc questionnaire to characterize the standard follow-up practice at the centers before the adoption of the remote service and evaluate the training provided and their experience with the service over the study period.

### Heart Connect™ application

**Figure 1:**
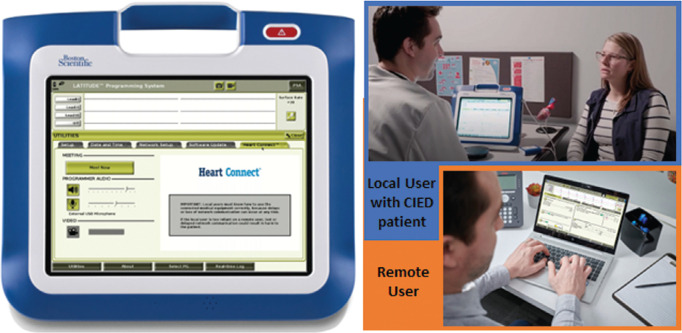
Remote support during cardiac implantable electronic device follow-up using the Heart Connect™ application on the Model 3300 Programmer. *Abbreviation:* CIED, cardiac implantable electronic device.

**Figure 2:**
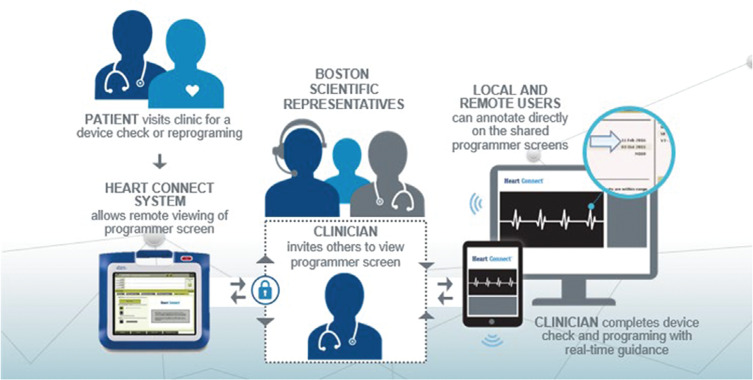
The Heart Connect™ application workflow.

The Heart Connect™ application is a data-sharing system intended to display and share physiological and other medical data from the Model 3300 Programmer **(Figure 1)**. The Heart Connect™ System provides health care providers and Boston Scientific personnel with the means to establish an online meeting and share the display of the programmer with individuals in a remote location. The local user (in this case, the health care provider) uses the application on the programming system to initiate a meeting with desired remote contacts (the IEAPs of the Remote Clinical Support team). Using optional audio and/or video, the user communicates with the remote user and initiates the video display sharing of the programmer screen **(Figure 2)**. Annotations can be made directly on the screens and shared between the local and remote users. With real-time guidance, the clinician can complete the device check and program as needed, but no programming or manual testing can be performed directly by the remote user. The system incorporates security measures for the protection of patient data and system integrity. The online meeting, including any images being transmitted, is encrypted to ensure patient information and device data are protected.

### Statistical analysis

Descriptive statistics are reported as mean ± standard deviation values for normally distributed continuous variables or medians with ranges in the case of skewed distribution. Categorical data are expressed as percentages.

## Results

### Participating centers and their standard practice of cardiac implantable electronic device follow-up before adoption of the remote service

**Figure 3:**
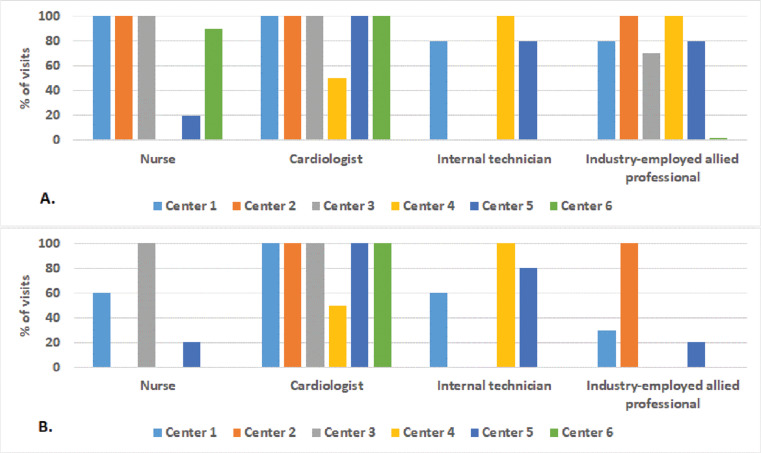
Health care personnel contributions within in-office device check during scheduled **(A)** and unscheduled **(B)** visits.

The remote technical support service using the Heart Connect™ application on the programmer was adopted in six Italian centers. The median number of patients with CIEDs followed up at the centers is reported in **Table 1**, along with the remote monitoring use and the average duration of visits. The involvement of each type of health care personnel during standard follow-up visits is shown in **Figure 3**. All centers typically made use of at least two different health care personnel during scheduled follow-up visits, with the most frequent combination being the involvement of both a cardiologist and a nurse or an internal technician. IEAPs were also frequently present in most centers. The distribution of health care personnel was different during unscheduled visits. The cardiologist, who appeared to be involved to the same extent, more rarely received support from nursing staff and IEAPs, who seemed to be present infrequently during unscheduled visits. In the case of events requiring an urgent check, the patient was frequently going directly to the clinic (approximately 23% of cases), out of a scheduled follow-up session. In addition to standard follow-up sessions, the centers also performed a median number of 15 (2–25) visits/year for the assessment of S-ICD eligibility, with a visit lasting an average of 17 ± 4 min and being usually performed by two operators. The IEAP seemed always present.

### Remote technical support

Overall, 20 operators received the remote support training. Survey questions on the training and documentation on the Heart Connect™ application are reported in **Table 2**. The level of satisfaction with the information received for the use of the application was generally high.

**Figure 4:**
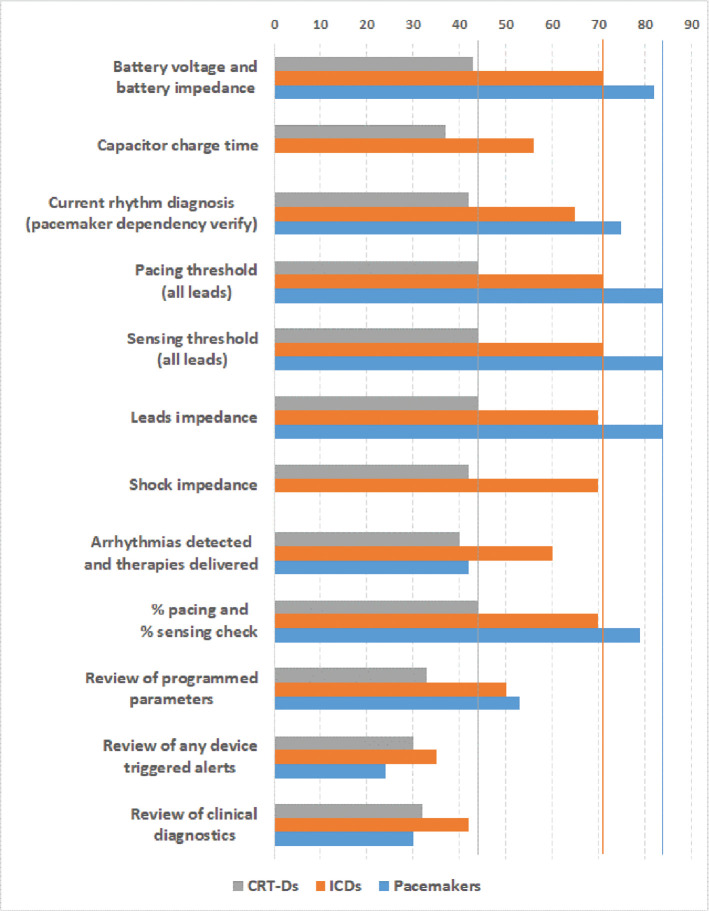
Cardiac implantable electronic device checks performed during the visits per device type (84 pacemakers, 71 implantable cardioverter-defibrillators, 44 cardiac resynchronization therapy defibrillators). In accordance with the work of Wilkoff et al.^6^

The network connection was preferably established using Wi-Fi in one center and a 4G cellular adapter in five centers. Online meetings were established during 205 follow-up visits and 3 sessions of S-ICD eligibility verification **(Table 3)**. Twelve (6%) of the follow-up visits were unscheduled. The remote connection was successfully established in 203 (98%) cases. In the remaining five cases, the connection was not established due to a temporary lack of Internet connection (three cases) and central server migration activities (two cases). In all cases of successful connection, the communication remained stable for the entire duration of the visit, except for six cases in which a loss of connection occurred (not restored in one case). The quality of the connection as well as the sound and video during two-way communication were rated good or excellent in >95% of sessions. Very comprehensive CIED checks and tests were performed during the visits **(Figure 4)**. During the three sessions of S-ICD eligibility verification, the remote support provided indications regarding the correct positioning of the ECG electrodes, the patient’s postures, the execution of the acquisitions, and the interpretation of the results. In all cases, the patient passed the test. Except for a single case of persistent loss of connection, the remote support allowed us to successfully complete all visits, without requiring additional medical or technical support. In most cases, the support was judged to be at least equally effective compared to the on-site support, without causing delays or organizational difficulties. Survey questions on the overall operator experience with the application and the remote technical support are reported in **Table 2**. In all phases of use, the application was judged to be user-friendly and effective. Most operators judged the remote service as an effective alternative to on-site support and appreciated the real-time support to facilitate the organization of follow-up in the center, with a positive impact on the duration of visits. Overall, the satisfaction with the remote support service was high.

## Discussion

We have demonstrated the feasibility and effectiveness of a remote support service as an alternative to the traditional on-site IEAP support during both scheduled and unscheduled follow-up visits for various CIEDs. Operators expressed positive opinions regarding the quality of remote interaction via the application and the level of technical support provided during the visits. Overall, the identified advantage of remote support was a reduction in visit times and a streamlined organization of unscheduled visits, making the process easier and more prompt.

Current recommendations^4^ emphasize the importance of CIED follow-up and monitoring activities to achieve significant goals, such as assessing and optimizing CIED system performance and safety, identifying and correcting any device abnormalities, and monitoring cardiac arrhythmias and physiological parameters. A survey conducted by the Heart Rhythm Society^7^ revealed that CIED follow-up checks already placed a substantial burden on physicians in 2010, with follow-up visits being the most frequent activity reported by electrophysiologists. These findings were further supported by a survey conducted by the European Heart Rhythm Association (EHRA), which indicated that real-world clinical practice requires significant resources for CIED follow-up, including time dedicated by specialized personnel such as cardiologists, nurses, internal technicians, and IEAPs.^8^ Given the increase in CIED implantations and limited availability of health care resources, there is a need to organize follow-up more efficiently. Moreover, there are CIED centers for which the continuity of on-site support is challenged, and the organization of follow-up visits is made more complex owing to their location. Remote monitoring of CIEDs has been proposed as an alternative to in-person visits to decrease the number of health care visits, enable earlier detection of actionable events such as arrhythmias, maintain safety, and improve survival.^9–14^ However, remote monitoring is not yet widespread throughout Europe,^15^ and in-office follow-up visits cannot be entirely eliminated.^4^ The follow-up of CIED devices can be particularly challenging when dealing with complex troubleshooting issues,^16,17^ which may require a high level of clinical and technical expertise. Paradoxically, the use of remote monitoring and an alert-based approach, where clinic visits are prompted only by the detection of actionable events, increases the complexity of in-person visits. Indeed, in cases of emergencies or clinical issues, higher-level expertise and longer visits are required to ensure optimal care.^8,18^

Consistent with previous findings,^8^ our centers reported that follow-up visits lasted approximately 15 min and involved at least two health care personnel, ie, a cardiologist and a nurse. The EHRA survey demonstrated that IEAPs were involved in approximately 20% of CIED follow-up visits across specialized centers in seven European countries.^8^ In our experience, centers reported more extensive use of IEAP support during scheduled CIED follow-up sessions. This discrepancy may be attributed to a different mix of CIEDs, differences in regulations and follow-up protocols among countries, or variations in center expertise. Notably, the significantly lower usage rate of IEAP support during unscheduled visits (<20%) indicates that technical assistance is often lacking in clinical practice just when it is needed most, during potentially challenging CIED follow-up visits involving complex troubleshooting issues. We observed that events requiring CIED checks (eg, when patients call or present at the hospital for symptoms or therapies) are often managed as urgent unscheduled visits, with device checks not being postponed to a scheduled session when availability of IEAP technical support is ensured.

In our experience, we have demonstrated the feasibility of a remote support service for CIED follow-up visits and the quality of the connection. Moreover, we tested the remote support service during various types of CIED patient visits, completing the visits without the involvement of additional medical or technical staff. The average duration of remote meetings was short, and the overall perception was that the visits were equivalent in duration—or even potentially shorter—and did not overburden the center with the adoption of remote support. While the observation period was relatively brief, the number of unscheduled visits was not insignificant, accounting for 6% of all follow-up visits, consistent with previous findings.^8,19^ In our series, the proportion of relevant conditions identified or actions and programming changes made during the visits was also considerable (29%), aligning with the proportion of actionable follow-ups found in the ATHENS multicenter registry.^20^ Even unscheduled or actionable follow-ups were effectively managed remotely, suggesting that the availability of real-time on-demand support can facilitate follow-up management at the center.

In summary, the operators expressed satisfaction with the remote support service and demonstrated interest in continuing to use it, particularly for managing unscheduled CIED follow-up visits, seeking prompt support, providing rapid feedback on device programming, and conducting efficient troubleshooting. However, longer observations are necessary to accurately determine the specific needs of each center, considering factors such as patient volume in follow-up, the range of CIEDs involved, the proportion of in-office visits conducted, and the expertise of the operators.

### Limitations

Our findings are subject to potential limitations. This study was conducted in a small number of centers over a relatively short period of time. Furthermore, the scope of the study was limited to CIEDs from a single manufacturer, which may restrict the generalizability of our results to other settings or systems. Moreover, this was purely a descriptive work with no direct comparison to standard practice with on-site IEAP support.

## Conclusions

Following a short initial training session, the use of Heart Connect™ for remote support during CIED follow-up demonstrated feasibility, effectiveness, and positive acceptance among operators. Through this study, it has been established as a viable alternative to traditional on-site IEAP support for both scheduled and unscheduled follow-up visits involving various types of CIEDs. Users expressed positive opinions and recognized the primary advantage of the remote technical support system—namely, the facilitation of more efficient and prompt organization of unscheduled visits.

## Figures and Tables

**Table 1:** Survey Questions on the In-clinic Follow-up Activity of the Study Centers

	n	CIEDs with Remote Monitoring Systems (%)	CIEDs from Boston Scientific (%)
Patients with CIEDs followed up in the center	2450 (450–4500)	15 (3–68)	32 (17–40)
- Pacemakers	1350 (200–3000)	8 (0–50)	30 (15–35)
- ICDs	650 (200–1300)	30 (15–90)	35 (24–42)
- CRT-Ds	275 (50–700)	33 (10–90)	38 (20–57)
		**CIEDs Without Remote Monitoring Systems**	**CIEDs with Remote Monitoring Systems**
Scheduled in-office visits for pacemakers (n/year)		2 (1–2)	1 (1–2)
Scheduled in-office visits for ICDs (n/year)		2 (2–2)	1 (1–2)
Scheduled in-office visits for CRT-Ds (n/year)		2 (2–2)	1 (1–2)
		**Scheduled Follow-up Visits**	
Average duration of a single visit (min)		15 ± 2	
Operators involved at the same time during a visit (n)		3 (2–3)	
		**Unscheduled Follow-up Visits**	
Average duration of a single visit (min)		19 ± 5	
Operators involved at the same time during a visit (n)		2 (1–2)	
Events requiring CIED check managed as urgent unscheduled visits (%)		23 (10–60)	

*Abbreviations:* CIED, cardiac implantable electronic device; CRT-D, cardiac resynchronization therapy defibrillator; ICD, implantable cardioverter-defibrillator.

**Table 2:** Survey Responses on Operator Experience with the Remote Technical Support Service Using the Heart Connect^™^ Application (N = 20)

Training			
Time for the training	35 ± 15 min		
The time for the training was	Insufficient: 0 (0%)	Adequate: 20 (100%)	Excessive: 0 (0%)
Information provided during the training was	Insufficient: 0 (0%)	Exhaustive: 9 (45%)	More than exhaustive: 11 (55%)
Need for additional information		0 (0%)	
The material provided was	Insufficient: 0 (0%)	Exhaustive: 9 (45%)	More than exhaustive: 11 (55%)
Need for additional material		0 (0%)	
Confidence with the system	Not confident: 0 (0%)	Somewhat confident: 4 (20%)	Completely confident: 16 (80%)
**Experience with the Application**	**Disagree**	**Neutral**	**Agree**
System connection and configuration were easy	0 (0%)	0 (0%)	20 (100%)
Accessory (speaker/headphones) connection was easy	0 (0%)	0 (0%)	20 (100%)
Searching for contacts to start a call was intuitive	0 (0%)	2 (10%)	18 (90%)
Starting a call was intuitive	0 (0%)	2 (10%)	18 (90%)
The remote interaction with the IEAP was effective	0 (0%)	1 (5%)	19 (95%)

Experience with the Service	Strongly Disagree	Disagree	Neutral	Agree	Strongly Agree
Remote support can effectively replace the on-site IEAP support for scheduled and unscheduled visits	0 (0%)	2 (10%)	0 (0%)	10 (50%)	8 (40%)
Real-time on-demand remote support facilitates follow-up management in the center	0 (0%)	0 (0%)	1 (5%)	4 (20%)	15 (75%)
The remote support allows to decrease the duration of visits	0 (0%)	2 (10%)	6 (30%)	7 (35%)	5 (25%)

Will continue to use the remote support in clinical practice	20 (100%)				
Preferred use of the remote service	- Scheduled follow-up: 6 (30%) - Unscheduled follow-up: 18 (90%) - S-ICD screening: 11 (55%) - MRI protection mode programming: 9 (45%)				
Remote service satisfaction	Very dissatisfied: 0 (0%)	Slightly satisfied: 0 (0%)	Moderately satisfied: 4 (20%)	Very satisfied: 9 (45%)	Completely satisfied: 7 (35%)

*Abbreviations:* CIED, cardiac implantable electronic device; CRT-D, cardiac resynchronization therapy defibrillator; ICD, implantable cardioverter-defibrillator; IEAP, industry-employed allied professional; MRI, magnetic resonance imaging; S-ICD, subcutaneous implantable cardioverter-defibrillator.

**Table 3:** Remote Technical Support Sessions (N = 203)

Remote Technical Support Sessions		
Attempted connections		208
Successful connection		203 (98%)
Visits completed successfully with remote support, n (%)		202 (97%)
- Need for additional support from IEAP, n (%)		0 (0%)
- Need to involve additional medical personnel, n (%)		1 (0.5%)

Session Supported Remotely	n (%)	Duration of Heart Connect™ Meeting (min)
Pacemaker follow-up	85 (42%)	6 ± 4
ICD follow-up	71 (35%)	8 ± 5
CRT-D follow-up	44 (22%)	7 ± 4
S-ICD eligibility test	3 (1%)	16 ± 9
Unscheduled follow-up, n (%)		12 (6%)
Relevant conditions identified or actions or programming changes done during the visit		All: 58 (29%) - Pacing and sensing parameters: 42 (21%) - Tachycardia detection and therapies: 8 (4%) - Elective replacement indicator: 5 (2%) - System failures: 2 (1%) - MRI protection mode activation: 1 (0.5%)

**Quality of the Meeting**	Poor	Unstable	Sufficient	Good	Excellent
Connection quality, n (%)	2 (1%)	2 (1%)	2 (1%)	48 (24%)	149 (73%)
Video quality, n (%)	0 (0%)	1 (0.5%)	5 (2%)	54 (27%)	143 (70%)
Audio quality, n (%)	3 (1%)	3 (1%)	6 (3%)	44 (22%)	147 (72%)

**Compared to the On-site IEAP Support, the Remote Support Was**	Worse	Equal	Better
Effectiveness, n (%)	2 (1%)	162 (80%)	39 (19%)
Duration, n (%)	12 (6%)	156 (77%)	35 (17%)
Ease of organization	1 (0.5%)	24 (12%)	178 (88%)

*Abbreviations:* CRT-D, cardiac resynchronization therapy-defibrillator; ICD, implantable cardioverter-defibrillator; IEAP, industry-employed allied professional; MRI, magnetic resonance imaging; S-ICD, subcutaneous implantable cardioverter-defibrillator.
